# A fabric-based soft hand exoskeleton for assistance: the ExHand Exoskeleton

**DOI:** 10.3389/fnbot.2023.1091827

**Published:** 2023-06-15

**Authors:** Juan C. Maldonado-Mejía, Marcela Múnera, Camilo A. R. Diaz, Helge Wurdemann, Mehran Moazen, Maria José Pontes, Marcelo Eduardo Vieira Segatto, Maxwell E. Monteiro, Carlos A. Cifuentes

**Affiliations:** ^1^Telecommunications Laboratory (LabTel), Electrical Engineering Department, Federal University of Espírito Santo (UFES), Vitória, Brazil; ^2^Department of Biomedical Engineering, Colombian School of Engineering Julio Garavito, Bogotá, Colombia; ^3^Bristol Robotics Laboratory, University of the West of England, Bristol, United Kingdom; ^4^Department of Mechanical Engineering, University College London, London, United Kingdom; ^5^Federal Institute of Espírito Santo (IFES), Serra, Brazil; ^6^School of Engineering, Science and Technology, Universidad del Rosario, Bogotá, Colombia

**Keywords:** hand exoskeleton, soft robotics, soft actuators, activities of daily living, assistive technologies

## Abstract

**Introduction:**

The rise of soft robotics has driven the development of devices for assistance in activities of daily living (ADL). Likewise, different types of actuation have been developed for safer human interaction. Recently, textile-based pneumatic actuation has been introduced in hand exoskeletons for features such as biocompatibility, flexibility, and durability. These devices have demonstrated their potential use in assisting ADLs, such as the degrees of freedom assisted, the force exerted, or the inclusion of sensors. However, performing ADLs requires the use of different objects, so exoskeletons must provide the ability to grasp and maintain stable contact with a variety of objects to lead to the successful development of ADLs. Although textile-based exoskeletons have demonstrated significant advancements, the ability of these devices to maintain stable contact with a variety of objects commonly used in ADLs has yet to be fully evaluated.

**Materials and methods:**

This paper presents the development and experimental validation in healthy users of a fabric-based soft hand exoskeleton through a grasping performance test using The Anthropomorphic Hand Assessment Protocol (AHAP), which assesses eight types of grasping with 24 objects of different shapes, sizes, textures, weights, and rigidities, and two standardized tests used in the rehabilitation processes of post- stroke patients.

**Results and discussion:**

A total of 10 healthy users (45.50 ± 14.93 years old) participated in this study. The results indicate that the device can assist in developing ADLs by evaluating the eight types of grasps of the AHAP. A score of 95.76 ± 2.90% out of 100% was obtained for the Maintaining Score, indicating that the ExHand Exoskeleton can maintain stable contact with various daily living objects. In addition, the results of the user satisfaction questionnaire indicated a positive mean score of 4.27 ± 0.34 on a Likert scale ranging from 1 to 5.

## 1. Introduction

Stroke is a leading cause of mortality and disability worldwide (Feigin et al., 2022). Common effects of stroke include communication impairments, balance and coordination deficits, reduced strength and motor control, and joint stiffness caused by spasticity (Murphy and Werring, 2020). In addition to these physical impairments, stroke survivors often experience dependence on others for activities of daily living (ADLs), altered mood, and impaired social interaction (Schultz et al., 1997), which can significantly diminish their overall quality of life.

Hand function loss is one of the most common impairments experienced by stroke survivors (Liu et al., 2018). Given that the hand is critical for performing many activities of daily living, including self-care, eating, writing, washing, and dressing, this impairment can significantly affect a person's independence and quality of life. The hand's complexity, with more than 20 degrees of freedom (DoFs) and a wide range of motion (RoM) in each joint, enables it to execute various movement patterns for grasping different objects (Kapandji, 1971). Thus, restoring hand function is essential for the rehabilitation of a stroke survivor.

The primary goal of stroke rehabilitation is to improve patients' quality of life and achieve the highest level of independence possible for each individual (Kelley and Borazanci, 2009; Good et al., 2011). This process typically begins with an evaluation of the patient's condition, which may include the use of outcome measures (Fetters and Tilson, 2018). Based on this assessment, the therapist will develop an individualized rehabilitation program that includes a series of exercises aimed at improving motor recovery and increasing hand and finger strength, dexterity, and range of motion (RoM).

However, hand rehabilitation is a long-term process that requires patience, persistence, and many repetitive exercise routines that involve interaction between the patient and therapist, making it a laborious and costly process. As a result, many stroke survivors discontinue therapy before achieving the maximum potential for hand function recovery (Mohammadi et al., 2018).

Advancements in technology have led to the emergence of hand exoskeletons, which aid in rehabilitation therapies and assist with activities of daily living (ADLs). Hand exoskeletons are soft robotics devices that are inspired by biological systems and are designed to be safer for humans (Hsiao et al., 2019). This technology uses soft and flexible components such as polymers (Rus and Tolley, 2015; Whitesides, 2018) to reduce size, complexity, weight, and cost (Ferguson et al., 2020). Furthermore, soft robotics has inspired the development of actuator designs for hand exoskeletons that perform movements kinematically similar to natural human joint movements (Whitesides, 2018). As a result, safe, lightweight, portable, and affordable devices have been developed.

Hand exoskeletons based on soft robotics have proven to be effective in recovering hand function (Aisen et al., 1997; Carmeli et al., 2011). In particular, these devices have drastically reduced the rehabilitation process's cost and the workload of therapists by enabling patients to perform intense repetitive movements (Wolf et al., 2006; Kutner et al., 2010).

Recently, the use of textile-based pneumatic actuation has been explored in the development of hand exoskeletons, leveraging the lightness, softness, flexibility, durability, and biocompatibility of fabrics (Sanchez et al., 2021; Fu et al., 2022). These properties are crucial for developing assistive devices (Boser et al., 2020; du Plessis et al., 2021). For example, researchers have shown that geometric variations in the textile structure can enhance the anisotropy, allowing for a wider range of motion and increased force generated by fabric-based actuators (Cappello et al., 2018a,b). Soft robotic gloves have been developed using flexible thermoplastic polyurethane (TPU) coated fabrics for bidirectional actuation (Yap et al., 2017), and multi-articular actuators and textile-based capacitance soft sensors have been incorporated into the next generation of gloves (Zhou et al., 2019). Furthermore, a study has been conducted to investigate the mechanical properties of various fabrics, leading to the design of a glove that can assist in thumb abduction, finger flexion, and extension movements (Ge et al., 2020). The development of textile-based hand exoskeletons shows promising results and is an emerging field that could have significant implications for stroke rehabilitation and ADL assistance.

Textile-based exoskeletons have shown great potential in assisting with activities of daily living (ADLs) for post-stroke patients, particularly in the execution of repetitive motions such as flexion, extension, and thumb abduction movements. These devices can generate the necessary force to grasp most objects commonly used in daily life, which are estimated to require a distal tip force of around 7.3 N, as most everyday objects weigh no more than 1.5 kg (Matheus and Dollar, 2010). For example, the devices presented by Zhou et al. (2019) and Ge et al. (2020) can exert forces of 37 and 47.9 N, respectively.

In addition, a study by Cappello et al. (2018b) demonstrated how their exoskeleton was able to assist in the rehabilitation therapy of spinal cord injury (SCI) patients through the Toronto Rehabilitation Institute Hand Function Test (TRI-HFT), which includes a manipulation test of 10 objects used in ADLs. However, while these devices have successfully provided the necessary force for grasping objects, their ability to maintain stable contact with various objects commonly used in ADLs has yet to be fully evaluated. For instance, Gerez et al. (2020) evaluated the grasping ability of a hybrid exoskeleton on 13 objects from the Yale-CMU-Berkeley object set, which is a collection of daily living objects that facilitates benchmarking in robotic manipulation and grasping (Calli et al., 2015).

Overall, while textile-based exoskeletons have shown promise in assisting with ADLs, further evaluation of their ability to grasp and maintain stable contact with various objects is needed to fully assess their effectiveness in daily life scenarios.

This paper builds upon the work of Ramos et al. (2022) by integrating textile actuators into an assistive device. In their study, Ramos et al. demonstrated that pleated textile actuators with a length of 16 cm and a width of 2 cm are capable of achieving a distal tip force of 9.18 ± 1.16 N, which is sufficient force to aid patients in manipulating various daily objects. Based on this finding, we present the development of a fabric-based hand exoskeleton, named the ExHand Exoskeleton, designed to assist stroke survivors with activities of daily living (ADL). Before its use on post-stroke patients, we experimentally validate the device on healthy subjects using the Anthropomorphic Hand Assessment Protocol (AHAP), a protocol developed by Llop-Harillo et al. (2019) for evaluating anthropomorphic robotic and prosthetic devices. The AHAP uses 25 objects from the Yale-CMU-Berkeley object set and quantifies the device's ability to hold the objects through eight relevant grasp types. Additionally, we evaluate the device's functionality through two outcome measures commonly used in the rehabilitation process of post-stroke patients. Finally, we evaluate the usability of the device through a questionnaire.

This document is structured as follows: Section 2 details the development and experimental validation of the ExHand Exoskeleton. Section 3 presents the evaluation results, and Section 4 compares our results with those of related work while highlighting advantages and limitations. Finally, we conclude and outline future work in Section 5.

## 2. Materials and methods

### 2.1. The ExHand Exoskeleton

As previously stated, this work presents the development and evaluation of the ExHand Exoskeleton. This section presents the exoskeleton actuators, the construction of the hand exoskeleton ExHand (its mechatronic system and its functionalities), and finally, the experimental validation of the exoskeleton.

### 2.2. Exoskeleton actuators

**Figure 1:**
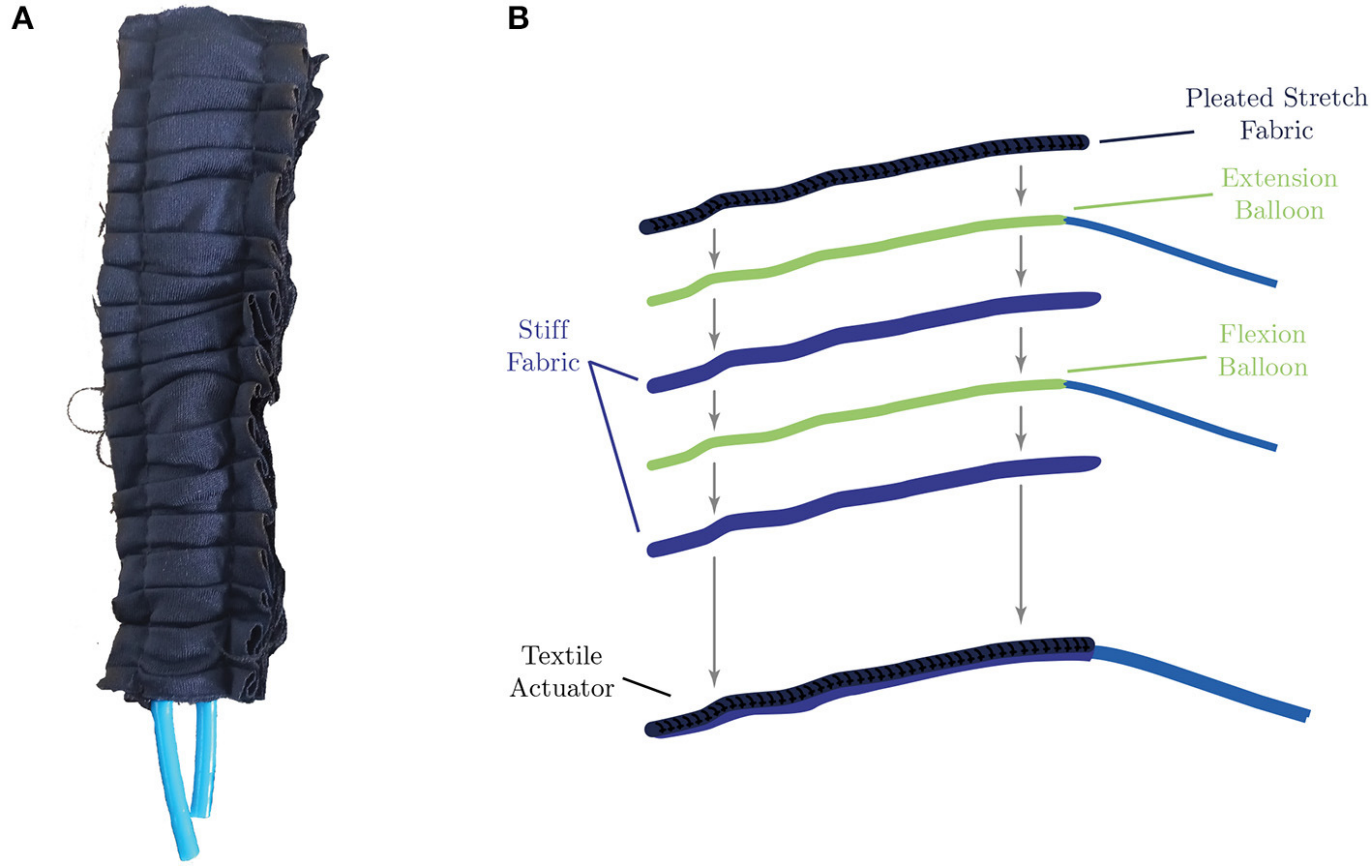
ExHand Exoskeleton actuator. **(A)** Textile-based actuator, composed of two balloons for flexion and extension movements and three layers of fabric, one layer of pleated stretch fabric, and two layers of stiff fabric. **(B)** Graphical representation of the textile-based actuator components.

Before using the textile actuators on the hand exoskeleton, their structure and operation are detailed. Elastic and inelastic fabric and thermoplastic elastomer (TPE) materials are used to construct the actuators. Specifically, a rigid fabric and an elastic fabric type, Lycra (Lycra-Nilon POWER ID-0019-056, Facol, Colombia), were used to construct the actuator. The construction process is done by creating a pocket from two layers of rigid fabric and adding a pleated elastic fabric on top. The fabric-based actuator comprises three layers of fabric (two layers of rigid material and one layer of plated stretch fabric) and two TPE balloons housed in the pockets generated by the fabric layers, as shown in Figure 1. Thus, flexion and extension movements are achieved by selective pressurization of the inner balloons.

### 2.3. Exoskeleton construction

**Figure 2:**
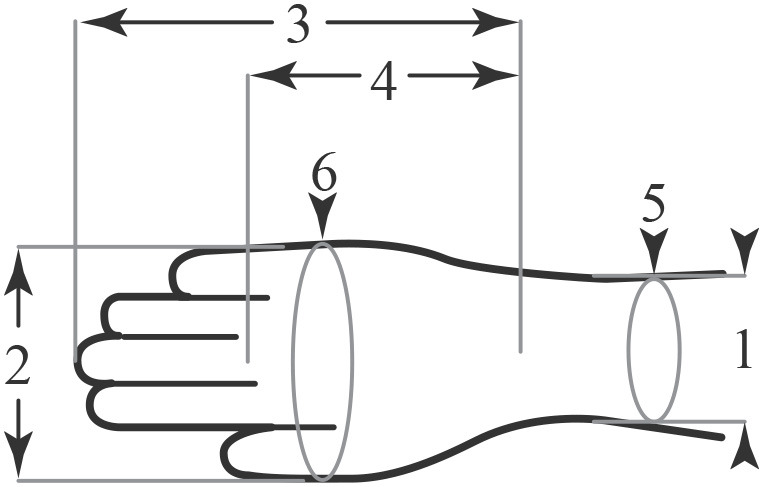
Human hand anthropometric measurements, illustration adapted from Chaurand et al. (2007).

The construction of the ExHand Exoskeleton is first carried out by searching for a suitable glove. The glove selection was based on the anthropometric measurements of the Colombian population (target population) and the glove sizing system used in Colombia, which uses two dimensions: metacarpal perimeter and hand length. Figure 2 and Table 1 present anthropometric measurements of the hand of the Colombian population divided by gender and percentiles, and Table 2 presents the glove sizes in Colombia.

**Table 1:** Anthropometric measures of the hand in the Colombian population, males (n = 1,315) and females (*n* = 785) between 20 and 59 years old (Chaurand et al., 2007).

**Gender**	**Female**			**Male**		
**Percentile**	**5**	**50**	**95**	**5**	**50**	**95**
1. Wrist width (cm)	4.48	4.93	5.43	5.00	5.58	6.05
2. Hand width (cm)	6.85	7.45	8.03	7.78	8.45	9.08
3. Hand length (cm)	15.43	16.60	17.95	16.83	18.30	19.90
4. Palm length (cm)	8.48	9.23	10.08	9.28	10.28	11.23
5. Wrist perimeter (cm)	13.53	14.68	16.10	15.20	16.53	17.98
6. Metacarpal perimeter (cm)	16.58	17.95	19.40	18.85	20.45	22.15

**Table 2:** Glove dimensions according to the Colombian sizing system (Rincón Becerra and Garc-a-Acosta, 2014).

**Glove size**	**Metacarpal perimeter of the glove (cm)**	**Hand length of the glove (cm)**
6	15.20	16.00
7	17.80	17.10
8	20.30	18.20
9	22.90	19.20
10	25.40	20.40
11	27.90	21.50

An anthropometric design requires adapting the products to 90% of the user population. For this reason, the most commonly used percentiles in ergonomic design are 5 (smaller people) and 95 (larger people), representing 90% of the population (Robinette, 2012). Therefore, the 95 percentile of the male is selected to ensure most users' comfortable use of the exoskeleton. Thus, glove size nine is the most appropriate for these measurements, as it has a metacarpal perimeter of 22.9 cm and a hand length of 19.2 cm, which is very close to the anthropometric measurements of the 95th percentile of the male (metacarpal perimeter of 22.15 cm and hand length of 19.90 cm).

**Figure 3:**
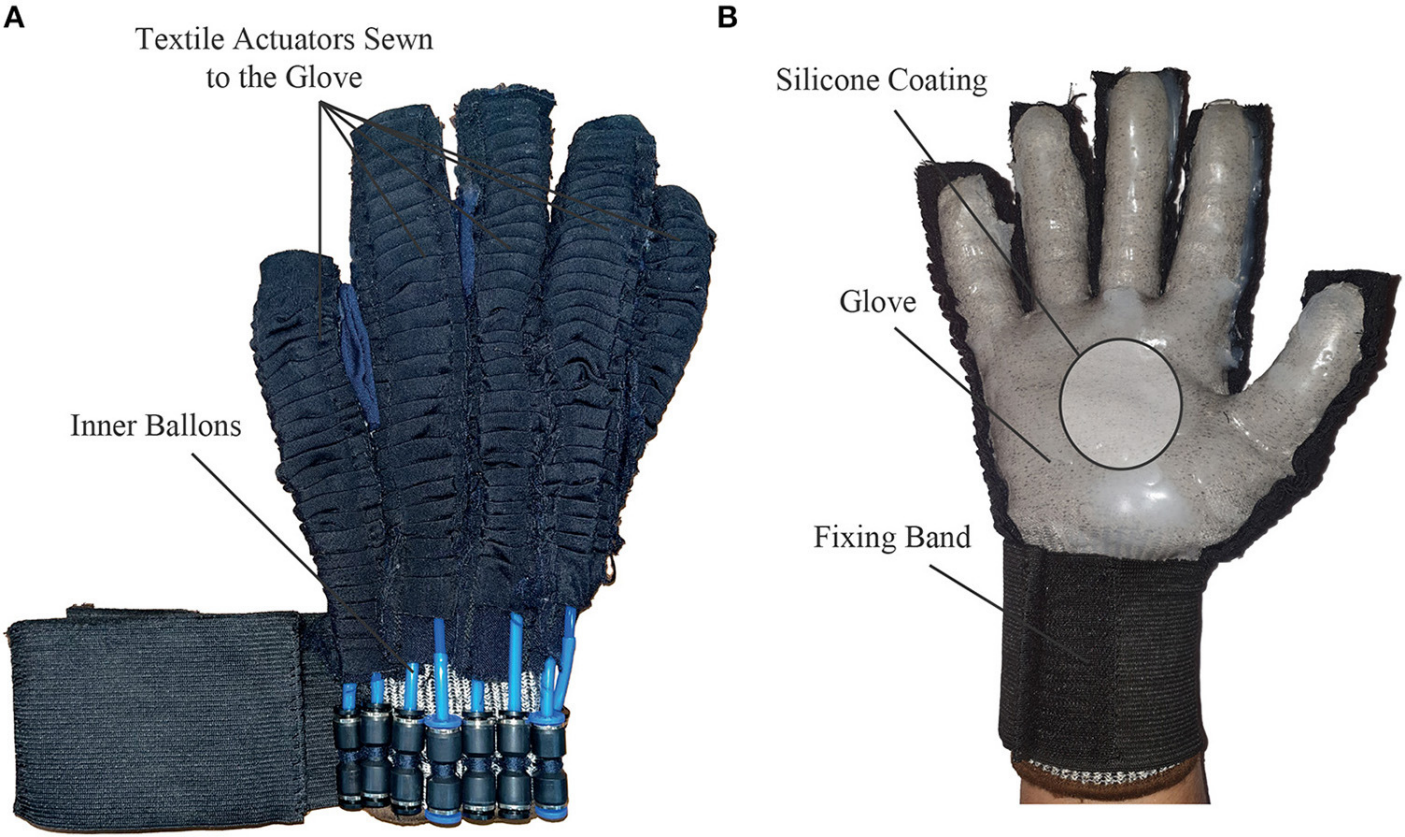
The ExHand Exoskeleton. **(A)** Dorsal side of the ExHand Exoskeleton shows the placements of the textile actuators on the glove. **(B)** Palmar side of the ExHand Exoskeleton, the coating of EcoFlex 00-30, and how the elastic band fixes to the user's wrist are shown here.

Once the glove is obtained, the actuators are placed. To facilitate the attachment of the actuators to the glove, a cutout is made to place them from the tip of the finger to the dorsal area of the glove. As a result, each actuator measured 13, 18.6, 19.6, 18.7, and 16 cm for the thumb, index, middle, ring, and little finger, respectively. Thus, the textile actuators were sewn around each finger from the tip of the finger to the dorsal part of the hand, and a silicone coating of Ecoflex 00-30 (Smooth-On, USA) was applied on the palmar area to generate a non-slip surface to improve grip. Finally, an elastic band with Velcro was added to fix the glove to the patient's wrist. The ExHand Exoskeleton is shown in Figure 3.

### 2.4. Mechatronics system

**Figure 4:**
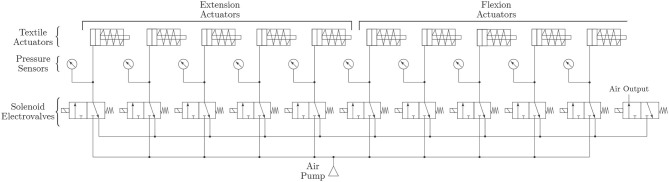
Schematic of the pneumatic system of the ExHand Exoskeleton.

The pneumatic system of the ExHand Exoskeleton is composed of an air pump ROB-10398 (Sparkfun Electronics, USA) of 32 psi of pressure. The ROB-10398 air pump can be used either as a vacuum pump or an air pump; in this case, the air pump is used for the ExHand Exoskeleton. For the selective pressurization of the balloons, a system of 11 solenoid electrovalves (Adafruit, USA) of three ways in two positions is implemented. Thus, 10 electrovalves control the flexion/extension movements performed by the selective pressurization of the internal balloons, and one electrovalve controls the air output. In addition, ten pressure sensors (MPX4250DP, NXP, Netherlands) have been added to measure the air pressure entering each of the inner balloons. Thus, air leakage due to over-pressurization is prevented, and the pressure can be adjusted to the user's requirements. The pneumatic schematic of the ExHand Exoskeleton is presented in Figure 4.

**Figure 5:**
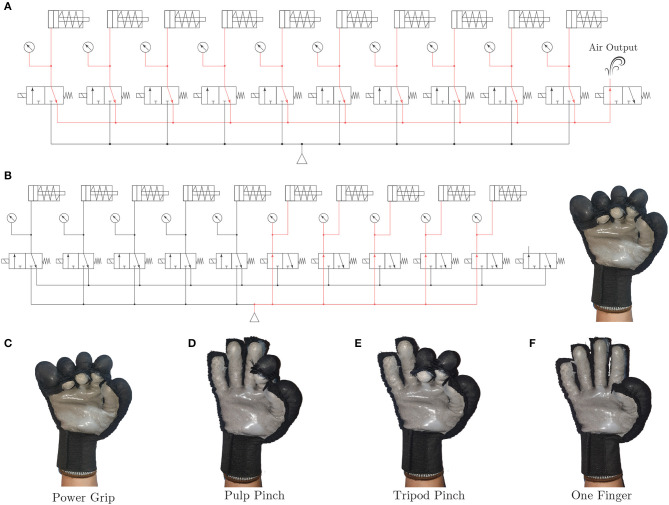
**(A)** For example, activating solenoid electrovalves for air release, the red lines indicate the airflow. **(B)** Activation of solenoid electrovalves to perform a power grip. Different grasp configurations of ExHand Exoskeleton: **(C)** Power grip, **(D)** pulp pinch, **(E)** tripod pinch, or **(F)** one finger.

The control of each internal balloon gives the exoskeleton the ability for the extension balloons to work simultaneously with the flexion balloons; this enables the exoskeleton to perform different combinations resulting in different types of grasp such as power grip, pulp pinch, tripod pinch commonly used in ADL, or actuate each finger separately if needed. Figure 5 shows some configurations.

A web interface was developed for the operation of the exoskeleton; in this interface, different modes of operation are established, for example, the extension of all fingers, different grips such as power grip, pulp pinch or tripod pinch, and depressurization of the system. Also, a configuration panel was added to adjust the pressure limits for each internal balloon as required by the user.

**Figure 6:**
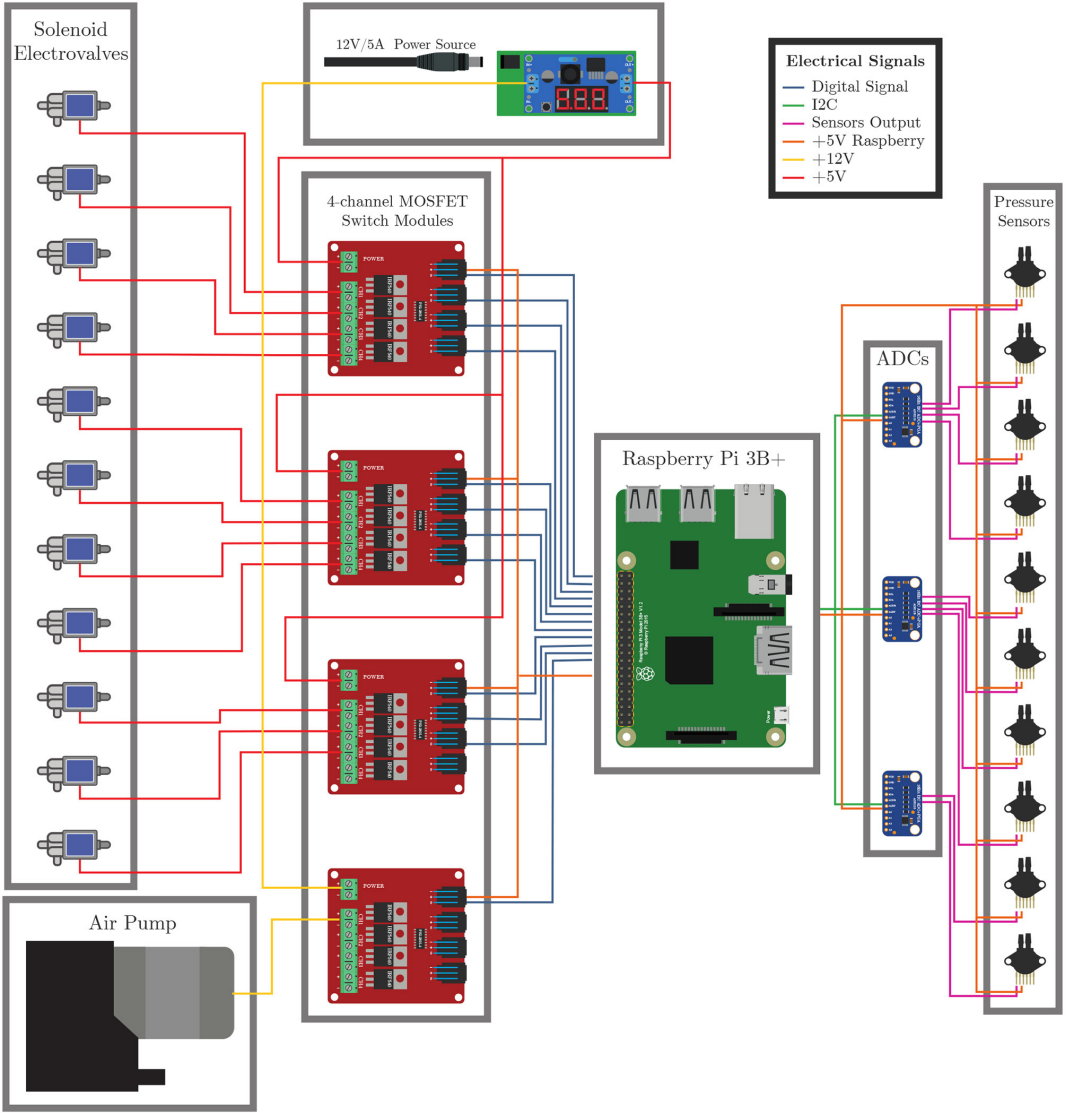
Electronic system and connections of the ExHand Exoskeleton.

Regarding the electronics system, 3 ADCs (ADS1115, Adafruit, New York, USA) are configured at 860 samples per second to read the pressure sensors' data. In addition, four 4-channel MOSFET switching modules were implemented as electric switches for the air pump and solenoid valves. Thus, as soon as a command is received from the web interface, the air pump, and solenoid valves corresponding to the requested motion are turned on, as shown in Figure 5A or Figure 5B. Once the pressure set by the user is reached, the air pump and solenoid valves are turned off to prevent over-pressurization. In the event of an air leak due to damage to the internal balloons, the air pump and corresponding solenoid valve will be kept until the user sends a different command from the web interface. All the processing and control of the device is performed by one single board computer (Raspberry Pi 3 B+) with the official operating system for Raspberry Pi systems based on Debian, Raspbian OS, and running Robot Operating System (ROS). In terms of consumption, the Raspberry Pi 3 B+ is sufficient to power the ADCs and pressure sensors, as each ADC consumes 5 V/150 μA, and each pressure sensor consumes 5 V/7.0 mA. As for the air pump (12 V, 1 A) and the solenoid valves (5 V, 220 mA), a separate 12 V/5 A power supply and a DC-DC voltage regulator (LM2596, DFRobot, Shanghai, China) set to 5 V are used. Finally, Figure 6 illustrates the electronic system and its connections for clarity.

### 2.5. ExHand Exoskeleton features

The study by Ramos et al. (2022) found that the textile-based actuators used to construct the ExHand Exoskeleton achieved a maximum distal tip force of 9.18 ± 1.16 N and a full bending time of 1.01 ± 0.33 s. Afterwards, benchtop experiments are performed to evaluate the time required to fully open and close the hand exoskeleton and the maximum force that can be exerted during grasping/holding.

#### 2.5.1. Time required to fully open and close

To measure the time required to open and close the exoskeleton completely, a volunteer was invited to done the exoskeleton while performing flexion and extension movements until the user's hand closed and opened as much as possible. The tests were recorded with a side camera, and the time was taken using a stopwatch from starting to pressurize the exoskeleton until the full flexion/extension movement was achieved.

#### 2.5.2. Exoskeleton maximum grasping/holding force

To evaluate the maximum grasping/holding force of the ExHand Exoskeleton, a 90 kg manual electronic dynamometer (Instruterm, Brazil) was used, and an experiment similar to that of Zhou et al. (2019) was performed, where grasping/holding force was evaluated as the bending force required to extend the pressurized flexion actuators of all fingers without the thumb. A 3D printing that included the flexion DoFs of the fingers was placed inside the glove.

### 2.6. Experimental validation with healthy users

#### 2.6.1. Participants

To evaluate the functionality and usability of the ExHand Exoskeleton, healthy users between 18 and 70 years of age, normal hand motor function, and the ability to perform gross and light gripping actions without discomfort or pain were included. The Ethical Committee in the Colombian School of Engineering Julio Garavito approved the study. All participants were informed about the scope and purpose of the study, and all participating individuals signed an informed consent form.

#### 2.6.2. Functional tests

As previously mentioned, the functionality of the ExHand Exoskeleton is evaluated through the AHAP and two outcome measures used in the rehabilitation process of post-stroke patients. For the development of the tests, the participants were instructed to relax their muscles and let the exoskeleton actuation assist the flexion and extension movements of the fingers. After reading and signing the informed consent, the exoskeleton is donned to the participant, and the test procedure is explained. Furthermore, the exoskeleton was controlled by an operator using the web application. The operator adjusts the air pressure value entering each inner balloon according to the user's hand to perform complete flexion and extension movements before starting the tests. Before grasping any object, the operator activates the extension movement, depressurizes the exoskeleton, and then activates the flexion movement according to the most appropriate grip for each object; once the object is released, the exoskeleton is depressurized again. All interventions were recorded using two cameras, one in front of the participant to capture a top view and the second on the side.

##### 2.6.2.1. Grasping performance test

The Anthropomorphic Hand Assessment Protocol (AHAP) (Llop-Harillo et al., 2019) was chosen to evaluate the ability of the textile-based ExHand Exoskeleton to grasp various everyday objects. This protocol defines a total score that quantifies the ability to perform everyday grasps using a set of internationally available objects. AHAP uses the YCB set of objects proposed by Calli et al. (2015), including 25 objects of daily life with different shapes, sizes, textures, weights, and rigidities. Within the objects are food items, kitchen items, tools, form items, and task items. Although the AHAP is focused on anthropomorphic hands for robotic and prosthetic applications, the results obtained by the protocol provide a baseline for comparison and a way to recognize possible improvements in the design of the devices, such as hand exoskeletons.

AHAP involves two non-grasp postures and eight different grasps: hook grip, spherical grip, tripod pinch, extension grip, cylindrical grip, diagonal volar grip, lateral pinch, and pulp pinch. In this case, the two non-grasp postures and one object associated with these postures were excluded since the evaluation of these postures would not provide revealing results for the analysis of grasping of the hand exoskeleton.

**Figure 7:**
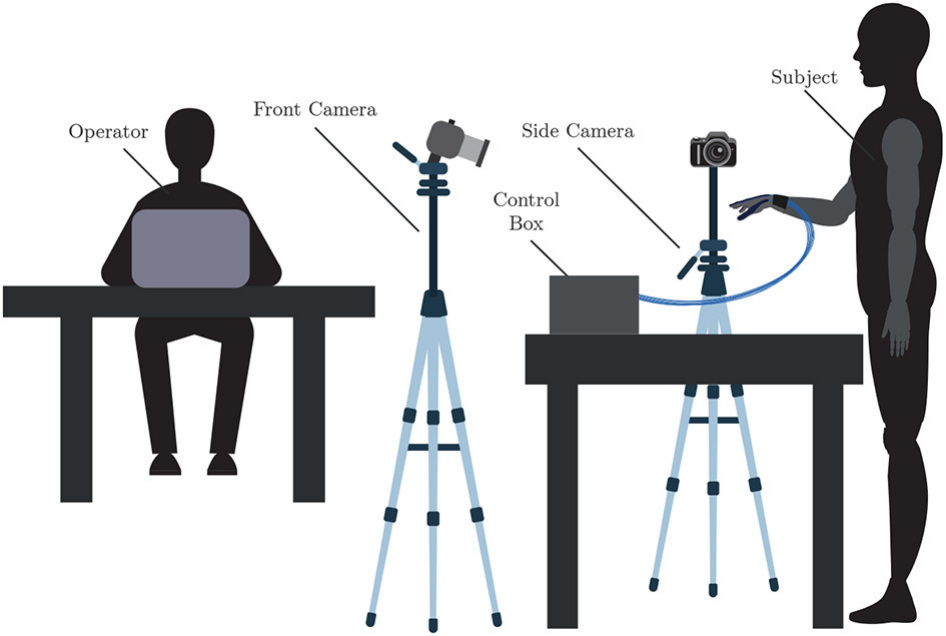
Experimental setup used to perform the AHAP, shows the position of the cameras, the subject, the operator, and the control box.

For the execution of the protocol, the participant must be standing and positioned near a table, as shown in Figure 7. Thus, the participant is instructed on the correct grasping posture for each object according to Llop-Harillo et al. (2019) and can practice with the object for 1 min. The objects are handed to the participant by the operator. Subsequently, the participant holds the grasp for 3 s. The participant naturally rotates the hand with low acceleration for the palm to point downwards (180°) and keeps the grip for another 3 s. Finally, the operator depressurizes the system to release the object.

The protocol is repeated three times for each object, and the score is divided as follows: the ability of the device to perform the grip correctly, similar to a healthy subject (*Grasping*) and the ability to hold the object without it moving (*Maintaining*). *Grasping* score and *Maintaining* score are scored from when the object is attempted to be grasped to when the object is released. In addition, *Grasping* is scored with values of 0, 0.5, and 1, being 1 if the grasping is completed with the correct grasping type, 0.5 if the grasping posture is different from the correct grasping type and 0 if the device cannot grasp the object. In the same way, *Maintaining* is scored with values of 0, 0.5, and 1, 1 if the object remains static while being held, 0.5 if the object moves and 0 if the object is dropped. The *Grasping* and *Maintaining* scores of the three objects of each type of grip are added together to obtain the final score for each grasp. Afterwards, a final score obtained from the two previous scores is calculated; this score is named the Grasping Ability Score (GAS) and quantifies the device's ability to perform all grasps. All scores are presented as a percentage. A GAS score of 100% means that the device can perform the different grasps in the same form as a healthy person (Llop-Harillo et al., 2019). To score the ExHand Exoskeleton, three external evaluators provided the *Grasping, Maintaining*, and GAS scores for each type of grasp using the video recorded during each test.

##### 2.6.2.2. Box and Blocks Test

Another test performed is the Box and Blocks Test (BBT). BBT is a common outcome measure used to assess the unilateral gross manual dexterity in various populations, including stroke survivors with mild-to-moderate deficits (Thompson-Butel et al., 2015; Kontson et al., 2017). This test complements the AHAP as its results provide information on how the use of the device may affect the user's dexterity to perform ADLs that require a precise grip. The BBT contains 150 wooden cubes of 2.5 cm of sides and a wooden box (dimensions 53.7 × 25.4 × 8.5 cm) divided into two compartments by a partition of 15.2 cm in height. The test consists of moving, one by one, the maximum number of blocks from one compartment of the box to the other within 60 s (Mathiowetz et al., 1985). The participant performed the test seated close to a table. The box was placed on the table on the participant's midline and oriented lengthwise, with the compartment containing the blocks oriented toward the hand evaluated (Mathiowetz et al., 1985). The final score is the number of blocks transferred from one box compartment to another in 60 s.

##### 2.6.2.3. Jebsen Taylor Hand Function Test

The Jebsen Taylor Hand Function Test (JTHFT) is another common outcome measure and has been used in clinical and research settings in different patient populations. The JTHFT assesses hand motor function through different ADL-related tasks (Jebsen, 1969). This consists of seven subtasks, including:

- Writing a 24-letter sentence.
- Turning over five cards of 7.6 × 12.7 cm (page turning simulation).
- Grasping five small objects (e.g., pennies, paper clips, bottle caps) and placing them in a container.
- Stacking five checkers.
- Simulated feeding.
- Moving five large empty cans.
- Moving five large heavy cans (450 g).

The participant must be seated close to a table to start the test. A stopwatch is used to record the time taken in each subtask. The total score is the sum of time taken for each subtask, where shorter times indicate better performance (Jebsen, 1969; Takla et al., 2018).

#### 2.6.3. Usability assessment

The Quebec User Evaluation of Satisfaction with Assistive Technology (QUEST) 2.0 has been applied to assess the participant's perception. This questionnaire was designed to assess people's satisfaction with assistive devices. QUEST includes the rating of 12 items using a 5-point Likert scale (1: not satisfied at all, 2: not very satisfied, 3: more or less satisfied, 4: quite satisfied, and 5: very satisfied) and is divided into three scores: *Device, Services*, and Total QUEST (Demers et al., 2000).

To identify the level of satisfaction or dissatisfaction of users when using the ExHand Exoskeleton, QUEST 2.0 was adapted to evaluate only six items corresponding to the *Device* score: Dimensions, weight, adjustment, safety, comfort, and effectiveness. The participants answered the questionnaire once the functional tests were completed.

## 3. Results

The development of the ExHand Exoskeleton with fabric-based actuation has been carried out. A glove with an approximate weight of 137 g and an approximate system weight (weight of the power supply, the air pump, the electrovalves, the pressure sensors, the ADCs, and the single board computer) of 971 g was obtained. In addition, ten participants (five males and five females, 45.50 ± 14.93 years old) performed the tests and completed the study successfully. All results were expressed as mean values with standard deviation.

First, the ExHand Exoskeleton achieves a full opening and closing time of 2.00 ± 0.35 and 3.47 ± 0.30 s, respectively. Furthermore, the exoskeleton's maximum grasping/holding force is 87.98 ± 1.55 N. Also, The air pressure values to perform complete flexion and extension movements are presented in Table 3. Table 3 shows that the extension values were slightly higher than the flexion values, indicating that greater force is required to open the hand than to close it. Furthermore, less air pressure was required to achieve complete flexion of the thumb and little finger with values of 4.63 ± 0.16 and 5.57 ± 0.09 psi, respectively.

**Table 3:** Air pressure values to reach complete extension and flexion movements in all fingers.

**Finger**	**Air pressure for extension movement (psi)**	**Air pressure for flexion movement (psi)**
Thumb	8.90 ± 0.16	4.63 ± 0.16
Index	9.95 ± 0.08	8.34 ± 0.28
Middle	10.45 ± 0.08	8.85 ± 0.37
Ring	9.95 ± 0.08	8.59 ± 0.22
Little finger	9.35 ± 0.24	5.57 ± 0.09

Second, Table 4 shows how the exoskeleton performs the different types of grasps with the objects of the AHAP and the scores obtained are presented in Table 5. Thus, the exoskeleton achieved a *Maintaining* high score in each of the different types of grasp. However, the *Grasping* score was only successful in the Hook grip and above 50% for the rest of the grasps, indicating that the ExHand Exoskeleton can hold the objects but not in the way indicated by the test. Finally, a GAS score of 80.80 ± 2.10% was obtained.

**Table 4:** The ExHand Exoskeleton performs the different types of grasps and objects of the AHAP.

**Grasp Type**	**Objects**		
Hook	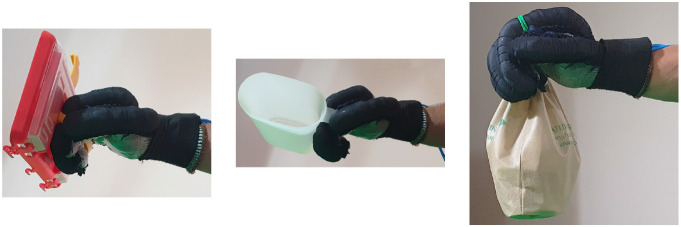		
Spherical grip	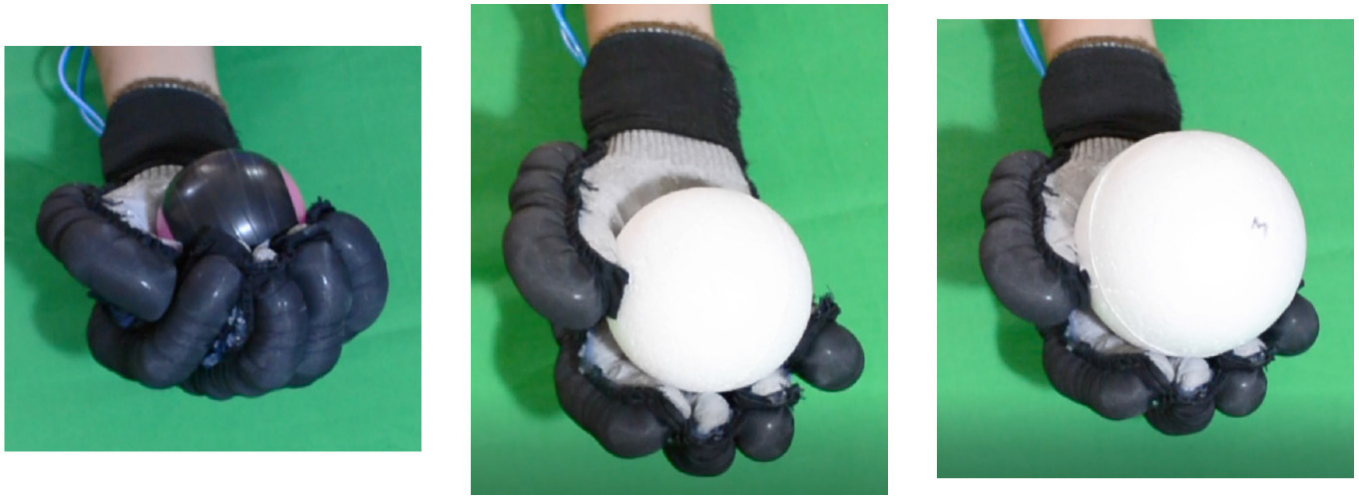		
Tripod pinch	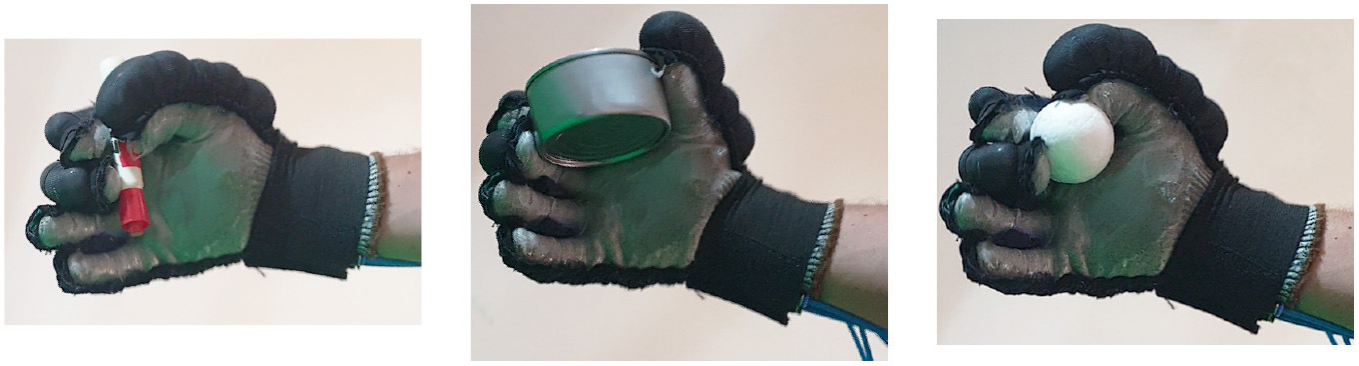		
Extension grip	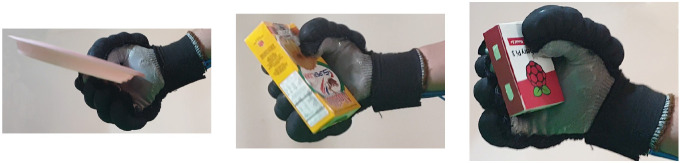		
Cylindrical grip	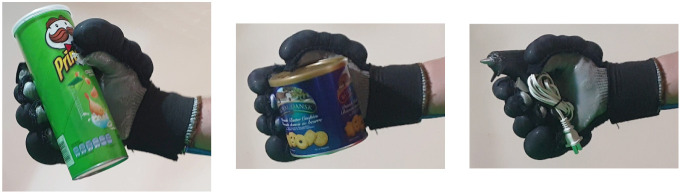		
Diagonal volar grip	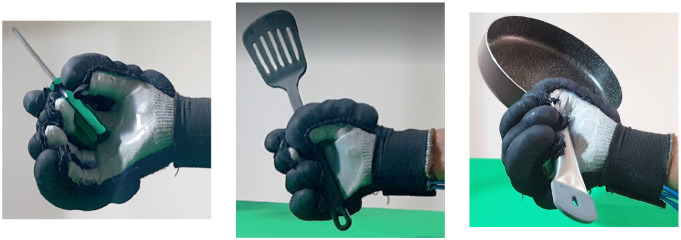		
Lateral pinch	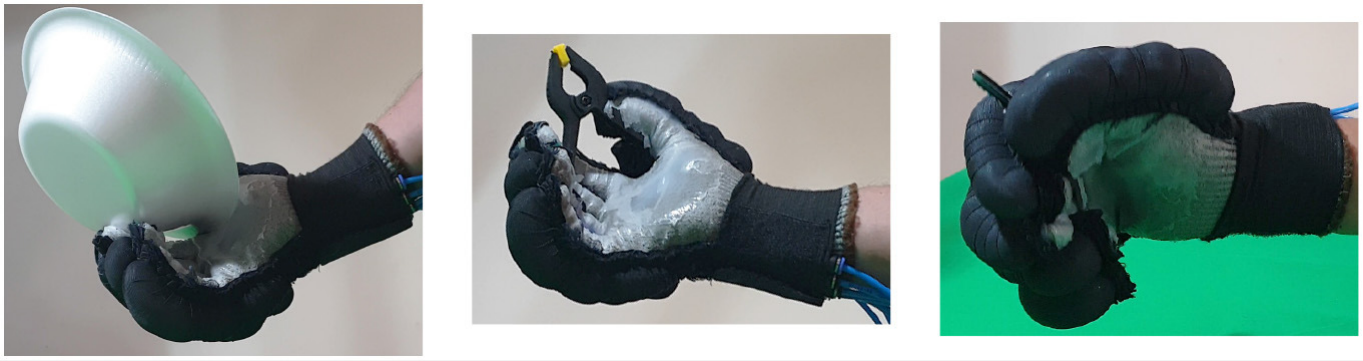		
Pulp pinch	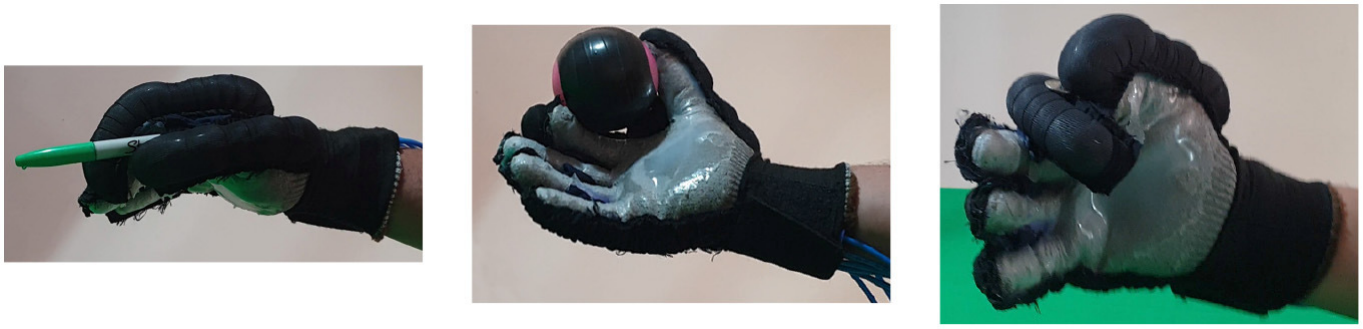		

**Table 5:** Grasping performance test scores divided into *Grasping, Maintaining*, and total GAS scores for the different types of grasp.

**Grasp type**	**Grasping score (%)**	**Maintaining score (%)**	**GAS score (%)**
Hook	100.00 ± 0.00	99.44 ± 0.48	99.72 ± 0.24
Spherical grip	73.33 ± 1.66	94.44 ± 4.81	83.89 ± 3.15
Tripod pinch	59.44 ± 3.15	93.33 ± 5.77	76.39 ± 1.34
Extension grip	63.88 ± 6.36	100.00 ± 0.00	81.94 ± 3.18
Cylindrical grip	68.33 ± 9.61	92.22 ± 4.19	80.28 ± 3.78
Diagonal volar grip	50.00 ± 0.00	93.89 ± 3.94	71.94 ± 1.97
Lateral pinch	61.66 ± 2.5	98.33 ± 1.44	80.00 ± 1.91
Pulp pinch	50.00 ± 0.83	94.44 ± 2.55	72.22 ± 1.20
Final score	65.83 ± 3.02	95.76 ± 2.90	80.80 ± 2.10

The gray color indicates the highest score in the grasping performance test.

Third, the number of blocks transferred during the BBT was presented in Table 6, indicating a total of 4.10 ± 0.57 blocks per minute using the exoskeleton. This value is minimally small compared to the 61.59 ± 7.75 blocks per minute without the exoskeleton.

**Table 6:** Score of BBT with and without the ExHand Exoskeleton.

**BBT score without Exoskeleton**	**BBT score with Exoskeleton**
61.50 ± 7.76	4.10 ± 0.57

Fourth, the JTHFT all subtasks scores and total performance time are shown in Table 7. The full performance time to complete de JTHFT with the ExHand Exoskeleton was 495.77 ± 31.38 s, and the better times using the exoskeleton were for the Writing Simulation and Feeding subtasks.

**Table 7:** Total performance time and scores of all subtasks of JTHFT with and without the ExHand Exoskeleton.

**Subtask**	**Performance time without Exoskeleton (s)**	**Performance time with Exoskeleton (s)**
Writing	12.74 ± 1.54	25.33 ± 3.18
Cards turning	5.18 ± 0.79	84.78 ± 7.77
Grasping small objects	5.02 ± 0.59	85.48 ± 8.03
Stacking checkers	6.48 ± 1.60	78.78 ± 4.56
Simulated feeding	9.47 ± 1.04	26.26 ± 3.84
Large empty cans	4.16 ± 0.64	93.21 ± 17.31
Large heavy cans	5.05 ± 1.17	101.94 ± 16.37
Total performance time	48.1 ± 5.43	495.77 ± 31.38

The times obtained in the JTHFT and the results of the BBT complement the AHAP results, as they demonstrate that the exoskeleton assists in the execution of various daily living tasks, such as feeding and writing. However, although the exoskeleton assists in ADLs, it performs these tasks more slowly than a healthy person.

Finally, the QUEST 2.0 scores are shown in Table 8. According to the scores, the device satisfaction means score for the participants was 4.27 ± 0.34 out of a maximum score of 5.

**Table 8:** Mean QUEST 2.0 scores.

**Item**	**Level of satisfaction**
Dimensions	4.20 ± 0.39
Weight	4.10 ± 0.44
Adjustment	4.40 ± 0.26
Safety	4.10 ± 0.37
Comfort	4.30 ± 0.34
Effectiveness	4.50 ± 0.26
Device satisfaction	4.27 ± 0.34

## 4. Discussion

A fabric-based soft hand exoskeleton for assistance in ADL was developed and evaluated, and a glove with a weight of 137 g and a system weight of ~971 g was obtained. Compared with other related devices, the weight of the ExHand Exoskeleton is similar to the glove of Ge et al. (2020), which weighs 128 g, or the glove of Yap et al. (2017) with a weight of 99 g. In terms of the maximum force exerted by the exoskeleton, a total of 87.98 ± 1.55 N was achieved. Comparing the force with similar devices, such as Ge's device, which achieved a force of 47.9 N (Ge et al., 2020), or the 37 N force of Zhou's device (Zhou et al., 2019), the ExHand Exoskeleton achieved a force above these devices. This difference in grasping/holding force with the other devices may be due to the method applied and to the silicone layer applied on the palmar side of the glove, which creates a non-slip surface. However, a better comparison would have to be made with the same materials and methods as those applied by Zhou et al. (2019) or Ge et al. (2020).

The experimental validation of the ExHand Exoskeleton in healthy users shows the exoskeleton successfully assists the participants in accomplishing all tasks. In the first instance, the pressure results indicate pressure values for extension movement between 8.90 ± 0.16 and 10.45 ± 0.08 psi and pressure values for flexion movement between 4.63 ± 0.16 and 8.85 ± 0.37 psi. In comparison with other similar fabric-based soft actuation devices, a similar value is found in the study of Yap et al. (2017), which requires 70 kPa (~ 10 psi) of input pressure to perform extension and flexion movements, contrary to other studies such as Cappello et al. (2018a,b) require a pressure of 172 kPa (~ 25 psi) or Ge et al. (2020) pressurizing their glove with 140 kPa (~ 20 psi).

In addition, the results of the AHAP show that the ExHand Exoskeleton can assist users in grasping objects of daily life with different shapes, sizes, textures, weights, and rigidities. It is evidenced by the *Maintaining* Score of 95.76 ± 2.90% in which a percentage higher than 90% was obtained for all types of grasps. However, it is observed that the lowest percentages were for the Tripod Pinch, Cylindrical Grip, and Diagonal Volar Grip with percentages of 93.33 ± 5.77%, 92.22 ± 4.19%, 93.89 ± 3.94%, respectively, due to the large and heavy objects such as a skillet, chips can or a tuna can, in which the contact of the glove with the object was not sufficient to maintain a stable contact. Therefore, the object moved, slipped or fell during the test. Therefore, although force tests are performed on the hand exoskeletons, combining them with grasping tests with different objects is important to verify that the devices assist and facilitate the grasping of different objects related to ADLs.

Also, a score of 65.83 ± 3.02% obtained in the *Grasping* score demonstrated the exoskeleton's incorrect grasping of different objects. Furthermore, the lowest percentages are obtained for pinch grasps, such as the tripod pinch, lateral pinch, or pulp pinch, similar to the findings of Cappello et al. (2018b) in the TRI-HFT administered to patients with SCI using the soft robotic glove.

Lastly, a GAS Score of 80.80 ± 2.10% is obtained, possibly related to the lack of movements such as abduction, adduction, and opposition to the thumb. Since the thumb represents the most important finger of the hand due to its ability to perform flexion, extension, and opposition, and more than 50% of types of grasps require thumb movements (Feix et al., 2009). Some exoskeletons have added active or passive actuators to add abduction/adduction movements to their devices. For example, Ge et al. (2020) implemented a textile actuator between the thumb and index finger to achieve thumb abduction. Li et al. (2019) placed actuators made of NinjaFlex 85A TPU between each finger to perform abduction/abduction movements. Gerez et al. (2020) developed a hybrid exoskeleton in which pneumatic chambers are added between each finger and an extra thumb to perform a secure and stable grip by increasing the contact area between the object and the glove.

Furthermore, the present study showed the performance of the ExHand Exoskeleton with 4.10 ± 0.28 blocks per minute for the BBT and a Total Performance Time of 495.77 ± 31.38 s in the JTHFT. Tables 9, 10 show similar studies to compare with other devices and analyze the performance of the ExHand Exoskeleton in the BBT and JTHFT.

**Table 9:** Box and Blocks Test (BBT) results in different studies found in the literature.

**References**	**Participants**	**BBT score**	
		**Without exoskeleton**	**With exoskeleton**
Zhou et al. (2019)	Two C4 and, one C5 SCI participants	3.67 ± 3.51	4.11 ± 3.17
Tran et al. (2020)	One C6 SCI participant	13	4
Thimabut et al. (2022)	20 stroke survivors	2.2 ± 0.6	8.60 ± 2.00
Dudley et al. (2021)	A stroke survivor	5	10
Radder et al. (2018)	65 older adults with different diseases and diagnoses	About 50	About 45
Polygerinos et al. (2015b)	A participant with muscular dystrophy	10	14

**Table 10:** Jebsen Taylor Hand Function Test (JTHFT) results in different studies found in the literature.

**References**	**Participants**	**JTHFT results**	
		**Subtask**	**Performance time without Exoskeleton (s)**	**Performance time with Exoskeleton (s)**

Tran et al. (2020)	One C6 SCI participant	Writing	28.20	27.70
		Cards turning	18.70	68.50
		Grasping small objects	34.30	113.00
		Stacking checkers	15.80	14.4
		Simulated feeding	14.10	65.20
		Large empty cans	9.70	*
		Large Heavy Cans	49.30	*
Radder et al. (2018)	65 older adults with differentdiseases and diagnoses	**Total performancetime without Exoskeleton (s)**	**Total performance time with Exoskeleton (s)**	
		About 78	About 95	
Van Ommeren et al. (2018)	Five chronic stroke patients	**Total performance time without Exoskeleton (s)**	**Total performance time with Exoskeleton (s)**	
		118.62	134.43	
Polygerinos et al. (2015a)	A healthy participant	**Subtask**	**Performance time with exoskeleton (s)**	
		Cards turning	44	
		Stacking checkers	26	
		Large empty cans	29	
		Large heavy cans	39	

On the one hand, compared to the study by Zhou et al. (2019), which also used a textile-based actuation glove, similar results were obtained in the BBT, showing that the ExHand Exoskeleton is slightly better by getting a lower standard deviation, it is also considered that our study was performed on healthy users so a better comparison will be made once the experimental validation of our device with pathological users is achieved. On the other hand, when comparing devices of different actuation, studies such as Tran et al. (2020), Radder et al. (2018), and Polygerinos et al. (2015a) found that the use of exoskeletons increases the time to complete the tests but assisted patients who failed to complete the test without the exoskeleton. Even so, the ExHand Exoskeleton showed a decrease in BBT and JTHFT performance. This could be related to the fact that the activities were performed with complete flexion and extension movements for every grasp, and the time required for the exoskeleton to perform the extension movement of 2.00 ± 0.35 s and the flexion movement of 3.47 ± 0.30 s, in addition to the deflation time which is about second. This can be seen in the times of the JTHFT subtasks, as the longer times are related to multi-object tasks, as opposed to the Writing subtask and Simulated Feeding subtask, as only one extension and one flexion movement were required to grasp a single object, a pen for the Writing subtask and a piece of cutlery for the Simulated Feeding subtask, which resulted in the shortest times of the ExHand Exoskeleton (times of 25.33 ± 3.18 and 26.26 ± 3.84 s, respectively) and similar to those presented by Van Ommeren et al. (2018) and Tran et al. (2020).

One way to improve the results in future evaluations is to create an internal balloon pressurization configuration that performs movements similar to those of the healthy person, considering that most of the grip types required partial extension or flexion movements instead of complete movements, thus decreasing the pressurization and depressurization times of the device. Also, more sensors such as bending, strain, or force sensors are considered for inclusion in future works to provide adequate grip force and posture feedback. Polymer optical fibers (POFs) are an emerging alternative for instrumentation in different applications. These sensors have been used to measure parameters such as angle, pressure, temperature, humidity, force, strain, and acceleration (Leal-Junior et al., 2019). In addition, POFs are immune to electromagnetic fields, have multiplexing capabilities, and are compact (Leal-Junior et al., 2019; De Arco et al., 2023), characteristics that make these types of sensors potentially suitable for use in soft hand exoskeletons. Another limitation is that the ExHand Exoskeleton does not recognize the human user's intention. Although a web interface is developed for easy operation of the device, future work will include a brain-computer interface (BCI), electroencephalography (EEG), or electromyography (EMG) signals as control signals to associate the patient's movement intention with the exoskeleton movement. Likewise, it is proposed to implement a control system using a combination of sensors to improve precision and thus promote better assistance.

Experimental validation also shows a mean positive score of 4.27 ± 0.34 for the QUEST 2.0 survey, i.e., an overall “quite satisfied” result. A value is similar to the studies conducted by Yoo et al. (2019) and Dudley et al. (2021) and the studies presented by Radder et al. (2019) and Tsai et al. (2019) in the System Usability Scale (SUS) questionnaire, another usability test. Surveys show people's acceptance of using these devices to assist or restore hand function, so using these surveys is recommended to provide researchers with information about user requirements and user satisfaction to compare subjectively with other devices (Pei et al., 2017; Yoo et al., 2019). Nevertheless, although healthy participants positively valued the ExHand Exoskeleton, users with anthropometric measurements lower than those of the glove mentioned that sometimes they did not perceive the grip of some objects, especially the smaller ones. Hence, it is considered the fabrication of various sizes of actuators according to different anthropometric measures and avoids the use of a glove. Furthermore, the involvement of clinicians and pathological users is needed to further validate the product's usability. Moreover, it is important to mention that the breathability of the glove was affected by applying a layer of silicone on the palmar side. Although the users were not uncomfortable, a study in post-stroke patients should consider procedures for device sanitization to avoid the risk of contamination by using the device in different users. Moreover, future works may involve the development of a device that leaves the users' palmar area free and thus avoids using a glove.

Finally, it is demonstrated that a textile-based exoskeleton, such as the ExHand Exoskeleton, can perform different grasps by evaluating its performance of 24 daily living objects of different shapes, sizes, textures, weights, and stiffness by achieving a score of 80.80 ± 2.10%, considering 100% means that the grips are performed in the same way as a healthy person. Likewise, it is highlighted that using 11 electrovalves to control the movement of each finger allows different grasp configurations as required by the user. Therefore, applying the AHAP (Llop-Harillo et al., 2019), a protocol for evaluating and comparing prostheses and robotic hands, is also a valuable tool in developing and comparing hand exoskeletons. Besides, the experimental validation of the exoskeleton with ten healthy subjects showed the repeatability of the study and similar results to the similar devices reported in the literature, confirming the device's suitability to perform a stable contact with a variety of daily living objects.

## 5. Conclusions and future works

The development and validation of a soft fabric-based hand exoskeleton assistance in ADL were presented. The results validate the ability of the application of the ExHand Exoskeleton to assist in grasping different types of objects used in ADLs. However, several challenges remain for the ExHand Exoskeleton to be addressed in future works, mainly the addition of low-cost, lightweight sensors; the development of actuators capable of different movements such as adduction and abduction, and opposition of the thumb; and lastly, the evaluation and validation of the device in stroke survivors.

## Data availability statement

The original contributions presented in the study are included in the article/supplementary material, further inquiries can be directed to the corresponding author/s.

## Ethics statement

The study was approved by the Ethical Committee in the Colombian School of Engineering Julio Garavito. All participants were informed about the scope and purpose of the study, and all participating individuals signed an informed consent form. The patients/participants provided their written informed consent to participate in this study.

## Author contributions

JM-M, HW, MMa, MMú, and CC contributed to the development and design of the hand exoskeleton and the performance of the study. JM-M performed the statistical analysis and wrote the first draft of the manuscript. All authors contributed to the manuscript revision, read, and approved the submitted version.
